# Happy people live longer because they are healthy people

**DOI:** 10.1186/s12877-023-04030-w

**Published:** 2023-07-18

**Authors:** Cai Feng Song, Peter Kay Chai Tay, Xinyi Gwee, Shiou Liang Wee, Tze Pin Ng

**Affiliations:** 1 Health and Social Sciences, Singapore Institute of Technology, 10 Dover Dr, Singapore, Singapore, 138683 Singapore; 2 Department of Psychological Medicine, Yong Loo Lin School of Medicine, National University of Singapore, Singapore, Singapore

**Keywords:** Happiness, Mortality, Positive affect, Longevity, Well-being

## Abstract

**Objectives:**

Higher levels of happiness are associated with longer life expectancy. Our study assessed the extent to which various factors explain the protective effect of happiness on all-cause mortality risk, and whether the association differs between older men and women.

**Methods:**

Using data from the Singapore Longitudinal Aging Studies (*N* = 6073) of community-dwelling older adults aged ≥ 55 years, we analyzed the association of baseline Likert score of happiness (1 = very sad to 5 = very happy) and mortality from mean 11.7 years of follow up. Cox regression models were used to assess the extent to which confounding risk factors attenuated the hazard ratio of association in the whole sample and sex-stratified analyses.

**Results:**

Happiness was significantly associated with lower mortality (*p* < .001) adjusted for age, sex and ethnicity: HR = 0.85 per integer score and HR = 0.57 for fairly-or-very happy versus fairly-or-very sad. The HR estimate (0.90 per integer score) was modestly attenuated (33.3%) in models that included socio-demographic and support, lifestyle or physical health and functioning factor, but remained statistically significant. The HR estimate (0.94 per integer score) was substantially attenuated (60%) and was insignificant in the model that included psychological health and functioning. Including all co-varying factors in the model resulted in statistically insignificant HR estimate (1.04 per integer score). Similar results were obtained for HR estimates for fairly-to-very happy versus fairly-to- very sad).

**Discussion:**

Much of the association between happiness and increased life expectancy could be explained by socio-demographic, lifestyle, health and functioning factors, and especially psychological health and functioning factors.

**Supplementary Information:**

The online version contains supplementary material available at 10.1186/s12877-023-04030-w.

## Translation significance

Higher levels of happiness are associated with longer life expectancy but the mechanisms of the relationship are not well investigated. Our results suggest that the presence of depression and self-perceived health and functioning explains almost all the relationship between happiness and mortality, and that happiness cannot be disentangled from a broader construct of psychological wellbeing and holistic health. Simply put, happy people live longer because happy people are healthy people. Policymaking should realistically adopt tangible strategies such as preventing or alleviating depression, improving physical and mental health, and psychological functioning and wellbeing.

*Do happy people live longer?* Happiness and joy are positive emotions and moods, in contrast to negative emotions and moods (such as anger and sadness). They are respectively positive affect (PA) and negative affect (NA) components of a broad construct of subjective wellbeing (SWB) —a third component being life satisfaction, pertaining to the cognitive evaluation of one’s own life [1]. The relationship between SWB and health and longevity has been the focus of a large number of studies in recent decades.

Studies have shown that SWB is associated with increased longevity [2–8]. Among the components of SWB, PA, rather than NA, appears to influence life expectancy [9, 10]. For example, using longitudinal data from a 22-year follow up cohort study, Gana, Broc [11] found that when all the SWB components (i.e., LS, PA and NA) were included in one model, only PA was significantly associated with longer life expectancy. There has also been some evidence suggesting that it is the frequency, rather than intensity, of PA that is more strongly related to well-being [12]. Given the importance of investigating and understanding the role of PA in promoting health and longevity, there are questions about the mechanisms and pathways through which happiness leads to health and longevity, as well as the question of gender differences. These have yet to be adequately addressed.

Socioeconomic, behavioral, mental, and physical health factors plausibly explain the link between happiness or SWB and longevity. They determine instrumental access to healthcare, avoidance of health risk, coping and resilience, and severity and outcomes of illnesses that eventually lead to premature death. Studies that have attempted to investigate these mechanistic links between PA and mortality have produced inconsistent results. While some studies have found that PA influences various health-related outcomes such as the avoidance of cardiovascular disease [13, 14], decreased symptoms and pain [15], and the adoption and maintenance of a healthy lifestyle [16], other studies have reported negative findings [17, 18]. Discrepant findings may largely be explained by “the variability in controlling for confounders across studies” [19]. Yet, even among studies that have controlled for similar sets of confounders, some divergence in results remain. For instance, Chei, Lee [19] in their analysis of data from 4,478 Singaporean men and women found happiness to be associated with lower mortality in both sexes after controlling for health, lifestyle, social and demographic variables. On the other hand, analyzing data from 719,671 women in the United Kingdom, Liu, Floud [20] concluded that happiness was not related to mortality after controlling for similar covariates which included self-rated health, various physical and mental health condition, and lifestyle and sociodemographic variables.

Apart from the differences in how happiness was measured and/or cultural differences, gender differences may explain the differing results. It is possible that the effect of happiness on mortality may be more potent for men than it is for women. A meta-analysis of studies showed that although subjective well-being was a protective factor for mortality, the protective effect was markedly stronger in men than women (Martín-María et al., 2017). The same conclusion was drawn for studies that used similar constructs such as optimism [21], life satisfaction, [22, 23], and enjoyment of life [24].

There are also limited numbers of studies investigating the link between happiness and mortality in Asian populations, which have socio-cultural views and perceptions about happiness that differ from the West [25–27]. Singapore, as a multiracial and multicultural Asian nation with a diverse mix of Chinese, Malay, Indian, and other races, holds the potential to offer valuable and pertinent insights for research on Asian populations. In this study, we investigated the association between happiness and all-cause mortality among middle-aged and older adults in Singapore, and examined the extent that this association can be explained by socio-demographic, lifestyle, health and functioning factors, and especially psychological health and functioning factors. We also examined whether the association between happiness and mortality differs by gender.

## Methods

### Study population

The present study used data from two combined population cohorts in the Singapore Longitudinal Aging Studies (SLAS), a population-based study on aging and health transitions. The SLAS recruited community-dwelling older adults aged ≥ 55 years, excluding individuals who were unable to participate due to severe physical or mental disability. Participants completed face-to-face interviews conducted by trained nurses at the participants’ homes, and clinical, physical and functional performance tests and blood draws were conducted at a local study site facility. A comprehensive range of demographic, psycho-social, behavioral, health-related data were collected from the first cohort in 2003–2005 (*N* = 2,804) and the second cohort in 2009–2010 (*N* = 3,270). Full details of the methodology of the two cohorts are available elsewhere [28–30]. The study was approved by the Institutional Review Board (IRB) of the National University of Singapore and all study participants provided informed consent. One case was excluded from analysis due to missing values and the analytic sample comprised of 6073 participants (62.8% female) for all-cause mortality.

### Measurements

#### Happiness

Happiness was measured via an approach that has been used in previous studies [20, 31–33], using a single item question. At baseline, participants were asked to respond to a question: “Do you feel that your life at present is…” with response options being “very happy”, “fairly happy”, “neither happy nor sad”, “fairly sad”, or “very sad”. Happiness was operationalized as both a continuous variable that ranges in values from 1 = very sad to 5 = very happy, and an ordinal variable with 3 categories (very or fairly happy, neither happy nor sad, very or fairly sad). We combined the “very” and “fairly” categories because of the low number of “very sad” participants.

#### Explanatory variables

*Sociodemographic* variables include questionnaire responses to age, gender, ethnicity (Chinese, Malay, Indian, Others), highest education (none, primary school, secondary/institute of technical education, post-secondary), housing type (1–2 room, 3-room, 4–5 room, others), and marital status (single, married, divorced/separated, widowed). *Instrumental social support score* for each participant was computed from their responses to questions on how frequent they receive phone calls and visits from friends or relatives as well as whether they report having a confidant.

*Lifestyle* variables include participant’s smoking habit (0 = never smoker, 1 = past smoker, 2 = current smoker, and alcohol intake (0 = never or less than once/week, 1 = sometimes [once or more/month but less than once/week], and 2 = Often [once or more/week]). Frequency of participation in multiple categories of physical, social and productive activities were measured on a Likert response scores (1 = “never or less than once a month”, 2 = “sometimes: more than once a month but less than once a week”, and 3 = “often: once or a week or more”), and participants’ responses were summed to derive their physical activity, social activity, and productive activity scores respectively [28]. A healthy lifestyle index was also created from combining responses to questions asking whether they watched what they eat, exercise regularly (i.e. 2–3 times a week), had good sleep, had time for leisure and relaxation, smoke or consume alcoholic drinks.

#### Cognitive health

The presence of cognitive impairment was assessed by a clinical screening and assessment protocol to diagnose mild cognitive impairment (MCI) and dementia. The procedures involved Mini Mental State Examination (MMSE) screening, Clinical Dementia Rating Scale (CDR) assessment, neurocognitive battery testing of cognitive deficits, and expert panel consensus diagnosis of MCI or dementia has been described in detail in previous publications [34].

#### Psychological health

Psychological health variables include participants’ history of mental illness (yes/no), if they were taking any antidepressant (yes/no), and the presence of depressive symptoms. The 15-item version of the Geriatric Depression Scale [GDS; [35]] was used to detect depressive symptoms (GDS ≥ 5) at baseline, and has been shown in older adults in Singapore to possess high criterion validity for determining the presence of major depression [36].

#### Physical health

For the purpose in this study, we included a superfluous number of related but distinct health indices commonly used in clinical research that are known important predictors of health outcomes including mortality but show low correlations to each other in this study.

The presence of *major chronic diseases* was determined by self-reported history of diagnosis or treatment, verified by drug names on medication packages, and/or clinical measurements or blood tests. These included diabetes (self-report or fasting blood glucose ≥ 5.6 mmol/L), hypertension (self-reported history or systolic blood pressure > 140 mm Hg or diastolic blood pressure > 90 mmHg), heart disease (self-report history including history of cardiac procedures), stroke (self-report), chronic obstructive pulmonary disease (FEV1/FVC < 0.70)) and chronic kidney disease (CKD defined by eGFR < 60 ml/min/1.73 m), and multi-morbidity (count of all chronic diseases).

The *frailty index* (FI) is a continuous measure of frailty that has been shown in many studies to predict mortality [37]. The FI variable is a continuous variable calculated based a cumulative deficit model, which takes into account the presence and/or severity of deficits in the form of presenting diseases, disabilities, and abnormalities from clinical examinations. Following a standard procedure for construction of index [38], FI was assessed in our study from 98 possible deficits across multiple systems, generating summed scores of binary variables (0 = no, 1 = yes) such as falls, hearing impairment, physical functioning limitation, poor self-rated health, obesity, history of medical conditions, and other health-related deficits [39]. The index is expressed as a fractional count measure: (number of known deficits /number of evaluable deficit) with values potentially varying from 0 to 1, higher fractional values denoting greater frailty [40]. Using a recommended minimum number of 30 deficits, the FI has been shown to be invariant to the number or type of deficits: the results yielded by the frailty index are consistent between different surveys which do not use the same deficits or the same number of deficits [38].

#### Functional limitations and disability

The participant’s mobility status was assessed and classified as immobile, wheelchair independent, walks with help of one person, or independent. A total count of dependencies on instrumental and basic activities of daily living (ADL) was used to assess functional disability. The SF12 health functioning status was assessed using the Physical Component Score (PCS) and the Mental Component Score (MCS). Participants’ self-rated global health was rated as 1 = excellent, 2 = very good, 3 = good, 4 = fair, 5 = poor.

### Mortality

The date and cause of death of the participants after baseline assessment was obtained by using their unique national registration identification card (NRIC) number for computerized record linkage with the National Death Registry of Singapore (NDR) via the National Disease Registry Office of the Ministry of Health Singapore. The NDR maintains all records of deaths that occurred in Singapore, and death registration is virtually complete in Singapore.

### Statistical analysis

Cox regression analysis was performed to estimate the association between happiness and mortality with survival time was measured in years from baseline to date of death or 31st March 2021. The contribution of sociodemographic and support, cognitive health, psychological health and functioning, physical health and functioning, and lifestyle factors in explaining the association between happiness and mortality was evaluated in separate sequential models. Model 1 was the base model, adjusted for age, gender, and ethnicity. Models 2 to 6 adjusted for different set of confounders —sociodemographic and support (Model 2), cognitive health (Model 3), psychological health and functioning (Model 4), physical health and functioning (Model 5), and lifestyle (Model 6). The final model, Model 7, was adjusted for all the confounding variables. To ascertain the percentage of the association between happiness and reduced mortality that may be explained by the different sets of confounders, we evaluated how much further the hazard ratio (HR) for happiness moved towards equipoise (i.e., HR = 1) in each model compared to the base model. For sensitivity analysis, we repeated the analyses using the nominal happiness variable (fairly-to-very happy versus fairly-to-very sad). To assess whether the association between happiness and mortality differs between men and women, we performed the Cox regression analysis separately for men and women with both the base model (Model 1) and the model adjusted for all potential confounders (Model 2). All analyses were performed using IBM SPSS v25. Values of *p* < 0.05 were considered significant.

## Results

At baseline, 1072 (17.8%) participants reported being very happy, 3652 (60.7%) were fairly happy, 1183 (19.7%) were neither happy nor sad, 87 (1.4%) were fairly sad, and 21 (0.3%) participants reported being very sad. Table 1 presents the demographic, social, and behavioral characteristics of the study participants by their level of happiness. Age, gender, ethnicity, education level, housing type, marital status, instrumental support score, physical activity score, social activity score, productive activity score and healthy lifestyle index were significantly associated with happiness both in unadjusted analysis and analysis adjusted for age, gender, and ethnicity. Participants’ health indices according to their level of happiness are presented in Table 2. Cognitive impairment, self-rated global health, depression, MCS, diabetes, heart disease, stroke, multi-morbidity, frailty index, and PCS were significantly associated with happiness in both unadjusted models and models adjusted for age, gender, and ethnicity.

**Table 1:** Demographic, social and behavioral characteristics of the study population (*n* = 6073) by continuum of happiness-sadness

	Variable		Level of Happiness							*Age, sex, ethnicity adjusted p*
		Full sample	Very Happy (*n* = 1072)	Fairly Happy (*n* = 3652)	Neither (*n* = 1183)	Fairly Sad (*n* = 87)	Very Sad (*n* = 21)	*F/X*^*2*^	*p*	
Demographic	Age	66.6 ± 7.8	65.7 ± 7.7	66.7 ± 7.7	67.1 ± 8.1	68.1 ± 8.7	65.4 ± 10.6	5.92	< .001	< .001
	Female sex	62.8 (3815)	60.7 (651)	64.3 (2348)	61.4 (726)	51.7 (45)	57.1 (12)	11.35	< .001	.022
Ethnicity	Chinese	89.9 (5454)	88.2 (945)	92.2 (3365)	85.6 (1012)	77.0 (67)	76.2 (16)	88.18	< .001	< .001
	Malay	6.0 (363)	5.8 (62)	4.5 (165)	9.8 (116)	11.5 (10)	19.0 (4)			
	Indian and other	4.1 (252)	5.9 (64)	3.3 (120)	4.6 (54)	11.4 (10)	4.8 (1)			
Highest education	No education	20.0 (1214)	16.2 (17.3)	20.5 (748)	21.8 (258)	19.5 (17)	23.8 (5)	144.28	< .001	< .001
	Primary	38.2 (2314)	27.1 (289)	40.1 (1461)	41.1 (485)	52.9 (46)	52.4 (11)			
	Secondary/ITE	30.7 (1859)	39.6 (423)	28.8 (1049)	29.5 (348)	20.7 (18)	19.0 (4)			
	Post-Secondary	11.1 (672)	17.1 (183)	10.6 (386)	7.6 (90)	6.9 (6)	4.8 (1)			
Housing type	1–2 room HDB	15.6 (937)	13.3 (142)	12.4 (450)	24.0 (281)	50.6 (42)	50.0 (10)	252.69	< .001	< .001
	3-room HDB	26.4 (1585)	23.0 (245)	26.7 (966)	29.1 (341)	16.9 (14)	40.0 (8)			
	4–5 room HDB	41.0 (2462)	40.6 (433)	43.6 (1577)	35.6 (416)	25.3 (21)	10.0 (2)			
	Others	17.1 (1026)	23.1 (247)	17.2 (622)	11.3 (132)	7.2 (6)	-			
Marital status	Single	7.6 (463)	7.1 (76)	6.1 (221)	12.3 (145)	10.3 (9)	28.6 (6)	141.28	< .001	< .001
	Married	68.6 (4157)	73.1 (783)	70.9 (2583)	59.3 (701)	55.2 (48)	33.3 ((7)			
	Divorced/Separated	4.6 (279)	4.3 (46)	3.8 (138)	6.3 (75)	16.1 (14)	14.3 (3)			
	Widowed	19.2 (1165)	15.5 (166)	19.3 (703)	22.1 (262)	18.4 (16)	23.8 (5)			
Social support	Live alone	7.2 (402)	7.4 (73)	6.6 (220)	8.3 (92)	9.5 (8)	21.1 (4)	10.14	.038	.07
	Instrumental support score (0–13)	25.5 (1550)	18.9 (203)	22.2 (811)	37.7 (446)	63.2 (55)	81.0 (17)	258.82	< .001	< .001
	(14–15)	36.4 (2208)	34.1 (366)	38.8 (1418)	32.5 (385)	20.7 (18)	9.5 (2)			
	(16–17)	38.1 (2315)	46.9 (503)	39.0 (1423)	29.8 (352)	16.1 (14)	9.5 (2)			
Lifestyle	Smoking: Non-smoker	79.8 (4844)	78.9 (846)	79.7 (2911)	80.8 (956)	79.3 (69)	95.2 (20)	5.38	.72	.53
	Past smoker	11.7 (712)	11.8 (127)	12.0 (437)	10.9 (129)	12.6 (11)	4.8 (1)			
	Current smoker	8.5 (517)	9.2 (99)	8.3 (304)	8.3 (98)	8.0 (7)	-			
	Alcohol: Never or rarely	92.2 (5558)	91.6 (976)	92.2 (3343)	93.0 (1090)	89.7 (78)	85.7 (18)	7.36	.50	.58
	Sometimes	4.3 (259)	4.6 (49)	4.4 (159)	3.8 (44)	3.4 (3)	4.8 (1)			
	Often	3.5 (210)	3.8 (41)	3.4 (122)	3.2 (38)	6.9 (6)	9.5 (2)			
	Physical activity score	2.3 ± 1.8	2.7 ± 1.9	2.4 ± 1.7	2.0 ± 1.6	1.6 ± 1.5	1.3 ± 1.1	26.64	< .001	< .001
	Social activity score	3.0 ± 2.4	3.5 ± 2.5	3.1 ± 2.4	2.6 ± 2.4	1.9 ± 1.8	2.1 ± 1.6	22.58	< .001	< .001
	Productive activity score	3.8 ± 1.9	4.1 ± 2.0	3.9 ± 1.9	3.5 ± 1.9	2.8 ± 1.9	2.7 ± 1.7	19.26	< .001	< .001
	Healthy lifestyle index	9.7 ± 1.97	10.1 ± 1.8	9.8 ± 2.0	9.3 ± 2.0	8.6 ± 2.3	8.0 ± 2.1	30.83	< .001	< .001

Figures are mean ± SD or % (n)

**Table 2:** Health indices of study population (*n* = 6073) by continuum of happiness-sadness

Health indices		Level of Happiness							*Age, sex,*
	Full sample	Very Happy (*n* = 1072)	Fairly Happy (*n* = 3652)	Neither (*n* = 1183)	Fairly Sad (*n* = 87)	Very Sad (*n* = 21)	*F/X*^*2*^	*p*	*ethnicity adjusted p*
Cognitive impairment	16.7 (985)	14.0 (147)	15.6 (555)	20.1 (229)	36.6 (30)	60.0 (12)	68.45	< .001	< .001
Depression	9.3 (563)	2.7 (29)	6.7 (219)	20.1 (239)	65.6 (57)	71.4 (15)	690.26	< .001	< .001
MCS score	54.4 ± 7.9	57.6 ± 6.0	54.8 ± 6.9	51.3 ± 9.9	42.5 ± 10.7	41.2 ± 9.0	169.24	< .001	< .001
Self-rated global health	3.1 ± .8	2.7 ± .8	3.2 ± .7	3.5 ± .7	3.8 ± .8	3.8 ± 1.0	169.95	< .001	< .001
Central obesity	6.6 (402)	5.6 (60)	6.6 (241)	7.4 (87)	9.2 (8)	9.5 (2)	4.07	.40	.43
Diabetes	16.3 (991)	15.1 (162)	15.5 (565)	19.2 (227)	23.0 (20)	33.3 (7)	17.49	.002	.026
Heart disease	12.2 (740)	10.5 (113)	11.0 (403)	15.3 (181)	32.2 (28)	28.6 (6)	38.50	< .001	< .001
Stroke	3.9 (235)	3.4 (36)	3.3 (121)	5.4 (64)	9.2 (8)	9.5 (2)	19.91	.001	.012
Chronic kidney disease	4.9 (295)	4.8 (51)	4.8 (175)	5.1 (60)	5.7 (5)	1 (4.8)	.32	.98	.99
COPD	25.1 (1162)	22.4 (178)	24.9 (711)	27.7 (244)	33.9 (21)	27.3 (3)	8.77	.07	.16
Number of chronic diseases	2.4 ± 1.5	2.2 ± 1.4	2.3 ± 1.5	2.6 ± 1.6	3.2 ± 1.5	3.5 ± 2.3	19.23	< .001	< .001
Frailty Index	.12 ± .07	.10 ± .06	.11 ± .07	.14 ± .08	.20 ± .09	.24 ± .10	108.47	< .001	< .001
PCS score	48.6 ± 7.2	50.7 ± 6.1	48.9 ± 6.8	46.2 ± 8.1	42.1 ± 10.3	39.4 ± 12.1	87.10	< .001	< .001

Depression: GDS ≥ 5 or history of depression or anti-depressant medication

Chronic kidney disease (eGFR < 60 ml/min/1.73 m; COPD: FEV1/FVC < 0.70

Self-rated global health: (1 = excellent, 2 = very good, 3 = good, 4 = fair, 5 = poor)

A total of 1337 (22%) deaths were observed from 71,331 person-years of follow up observation: 190 (17.7%) deaths among very happy participants, 786 (21.5%) deaths among fairly happy participants, 298 (25.2%) deaths among neither happy nor sad participants, 36 (41.4%) deaths among the fairly sad participants, and 10 (47.6%) deaths among the very sad participants. Table 3 presents the mortality rate by the level of happiness for the whole sample and for men and women separately. In Cox regression models (Table 4), happiness was significantly associated with mortality in the base model (*p* < 0.001) adjusted for age, sex and ethnicity: HR = 0.85 per integer score and HR = 0.57 for fairly-or-very happy versus fairly-or-very sad. The strength of association was modestly attenuated (by 33.3%) in models that included socio-demographic and support factor, lifestyle factor or physical health and functioning factor, but remained statistically significant (HR = 0.90 per integer score). However, the HR estimate (0.85 per integer score) remained virtually unaltered in the model that included cognitive health factor. On the other hand, the HR estimate (0.94 per integer score) was substantially attenuated (by 60%) and was insignificant in the model that included psychological health and functioning. In the final model that included all co-varying factors, there was no statistically significant association with mortality (HR = 1.04 per integer score). In this model, adjusted for all confounders, age, gender, ethnicity, housing type, instrumental social support score, self-rated health, diabetes, COPD, CKD, heart disease, IADL dependency, social activities score, smoking frequency, and alcohol intake frequency were significantly associated with mortality. Similar results were obtained with HR estimates of fairly-to-very happy versus fairly-to-very sad.

**Table 3:** Mortality rates (*n* = 6073) by continuum of happiness-sadness

		Level of Happiness				
	Full sample	Very Happy	Fairly Happy	Neither	Fairly Sad	Very Sad
Whole sample						
No. of participants	6073	1072	3652	1183	87	21
Person-years of observation	71,331.0	12,845.0	43,349.1	13,358.2	903.5	168.6
No. of deaths	1337	190	786	298	36	10
Deaths/1000 p-y	18.7	14.8	18.1	22.3	39.8	59.3
Crude HR (95%CI)	NA	0.23 (0.12–0.43)	0.28 (0.15–0.52)	0.35 (0.19–0.66)	0.63 (0.31–1.28)	1
Men						
No. of participants	2258	421	1304	457	42	9
Person-years of observation	25,162.8	4886.6	14,596.4	4908.2	425.3	62.6
No. of deaths	695	99	403	157	21	6
Deaths/1000 p-y	27.6	20.3	27.6	32.0	49.4	95.8
Crude HR (95%CI)	NA	0.18 (0.08–0.42)	0.25 (0.11–0.56)	0.30 (0.13–0.67)	0.46 (0.19–1.14)	1
Women						
No. of participants	3815	651	2348	726	45	12
Person-years of observation	46,168.2	7958.4	28,752.7	8449.9	478.2	106.0
No. of deaths	642	91	383	141	15	4
Deaths/1000 p-y	13.9	11.4	13.3	16.7	31.4	37.7
Crude HR (95%CI)	NA	0.29 (0.11–0.79)	0.34 (0.13–0.90)	0.43 (0.16–1.17)	0.83 (0.28–2.51)	1

**Table 4:** Association of happiness with all-cause mortality (deceased, *n* = 1337; alive, *n* = 4736) after adjusting for different sets of confounders

Analytic Models		HR per integer score of happiness scale (1–5)	Explained, %	HR for fairly or very happy vs fairly or very sad	Explained, %
Model 1	Age, gender, ethnicity	***0.85 (0.78–0.93)		**0.57 (0.41–0.79)	
Model 2	Model 1 + socio-demographic and support (education, housing type, marital status, instrumental social support score)	*0.90 (0.83–0.98)	33.3	*0.68 (0.49–0.93)	25.6
Model 3	Model 1 + lifestyle (smoking, alcohol, physical, social, and productive activity scores, healthy lifestyle index)	**0.90 (0.83–0.97)	33.3	**0.64 (0.48–0.87)	16.3
Model 4	Model 1 + cognitive health (cognitive impairment)	***0.85 (0.79–0.92)	0	**0.60 (0.44–0.82)	7.0
Model 5	Model 1 + psychological health and functioning (depression, MCS score, self-rated global health)	0.94 (0.85–1.03)	60.0	0.75 (0.53–1.05)	41.9
Model 6	Model 1 + physical health and functioning (Frailty index, BMI, diabetes, heart disease, stroke, CKD, COPD, arthritis, mobility, IADL dependency, PCS score, multimorbidity)	*0.90 (0.83–0.98)	33.3	**0.65 (0.48–0.88)	18.6
Model 7	All predictors	1.04 (0.94–1.14)	100	0.84 (0.58–1.23)	62.8

Happiness scale: 1 = very sad to 5 = very happy

Dependent variable: time until death

^*^p < .05

^**^p < .01

^***^p < .001. *HR* hazard ratio, *CI* confidence interval

### Sex differences

Happiness was significantly associated with mortality for both men and women in the age-and-ethnicity-adjusted model (Model 1) but not when the model was adjusted for all potential confounders (Model 2). The results are presented in Table 5. Age, housing type, COPD, diabetes, heart disease, stroke, self-rated health, and alcohol intake frequency were significantly associated with male mortality, while age, ethnicity, housing type, self-rated health, COPD, CKD, diabetes, heart disease, IADL dependency, PCS score, social activities score, and smoking frequency were associated with female mortality.

**Table 5:** Cox regression hazard ratios for the association between happiness and mortality by gender

Groups	n	Deaths/1000 person-years	Hazard ratio (95% confidence interval)							
			Per integer happiness score				Fairly or very happy vs Fairly or very sad			
			Model 1		Model 2		Model 1		Model 2	
Overall	6073	18.74	0.85 (0.78–0.93)	< .001	1.04 (0.94–1.14)	.50	0.57 (0.41–0.79)	.001	0.84 (0.58–1.23)	.37
Men	2258	27.62	0.82 (0.74–0.92)	.001	1.00 (0.88–1.15)	.97	0.56 (0.37–0.85)	.006	0.95 (0.58–1.57)	.85
Women	3815	13.91	0.88 (0.77–0.99)	.04	1.08 (0.93–1.25)	.33	0.53 (0.31–0.91)	.02	0.85 (0.45–1.60)	.62

Model 1 was adjusted for age, gender, and ethnicity. Model 2 was adjusted for age, gender, ethnicity, education, housing type, marital status, instrumental social support score, cognitive impairment, depression, MCS score, self-rated global health, frailty index, BMI, diabetes, heart disease, stroke, CKD, COPD, arthritis, mobility, IADL dependency, PCS score, multimorbidity, smoking frequency, alcohol intake, physical activities score, social activities score, productive activities score, and healthy lifestyle index

*HR* hazard ratio, *CI* confidence interval

## Discussion

We found in this study of Asian middle-aged and older adults that happiness was associated with decreased mortality. However, the association could be entirely attributed to a combination of sociodemographic and support, cognitive health, psychological health and functioning, physical health and functioning, and lifestyle factors. Using a semi-continuous measure of happiness (1 to 5), these factors cumulatively explained virtually 100% of the protective effect of happiness. Psychological health and functioning factors, which includes the presence of depression, mental health score, and self-rated health, accounted for the highest proportion (60%) of the link between happiness and reduced mortality. On its own, it rendered the happiness-mortality association insignificant after its inclusion in the model. Physical health and functioning-related factors accounted for a third of the protective effect of happiness (33%) and its impact appears comparable to socio-demographic and support, and lifestyle factors.

Our findings mirror those by some previous studies, for example, Liu, Floud [20] and Barger, Broom [31], and run counter to other studies, for example Gana, Broc [11], Kimm, Sull [41], Tamosiunas, Sapranaviciute-Zabazlajeva [42], and Zaninotto and Steptoe [3], and Chei, Lee [19]. This is in large parts due to the varying extent to which confounding risk factors were taken into account in different studies. In studies which control for a limited number of pertinent confounding risk factors, residual confounding may still explain an observed significant association in multivariable analyses. Uniquely, in our study, we controlled for a superfluous number of potential confounding variables yet avoiding over-adjustment. Specifically, in regard to physical and mental health as a major confounding risk factor, the content measurement of this holistic construct is seldom complete. Virtually all the measures of mental or physical health in our study showed very low inter-correlations, (*r* = -0.004 to 0.51) suggesting they measure some overlapping but also different aspects of mental and physical health (see Supplementary Table S1).

There is potentially a great overlap between happiness and psychological health and functioning which account for the observation that model inclusion of psychological health and functioning attenuated by 60% the mortality HR estimate. This result is congruent with the findings by Liu, Floud [20] who found that happiness was no longer significantly associated with all-cause mortality once the model was adjusted for self-rated health. In this study, the correlation between happiness score and a combined principal component analysis (PCA) factor score of psychological health and functioning (depression, MCS score, self-rated global health) was 0.43. Happiness is intrinsically related to psychological wellbeing and mental health, and the measurement of happiness as a distinct positive affect construct is elusive. Our results suggest that it cannot be disentangled from a broader construct of psychological wellbeing and holistic health. It is therefore unsurprising that the presence of depression and self-perceived health and functioning should explain almost all the relationship between happiness and mortality.

Contrary to what the current literature may suggest, we found no evidence of gender differences. Previous studies had found that the protective effect of related measures of wellbeing such as SWB [5], life satisfaction, [22, 23], optimism [21], and enjoyment of life [24] on mortality is more pronounced in men than in women.

The inconsistency in the current literature could also be dominantly due to the differences in the use of different positive psychological wellbeing constructs including ‘happiness’, and how happiness as a PA construct is operationalized across studies. It is interesting to note that studies reporting similar results to ours generally also utilized a single-item approach in operationalizing happiness [20, 31]. On the other hand, studies with results divergent to ours tend to use derived measures of happiness by combining multiple items [11, 19, 41–43]. Some research indicated that single-item measures can perform very similarly to multi-item measures [44, 45], even across multiple large cohorts (Cheung and Lucas, 2014). However, whether this applies to single-item or multi-item measures of happiness remain unclear and unresolved. More research is needed in this area to elucidate and reconcile these inconsistencies.

### Implications of study findings

Simply put, our results suggest that *happy people live longer because happy people are healthy people.* Policymaking should realistically adopt tangible strategies such as preventing or alleviating depression, improving physical and mental health, and psychological functioning and wellbeing. This promotes both healthy and happy longevity.

### Strengths and limitations

In this prospective cohort study of a large population-based sample of community-dwelling older adults with complete ascertainment of mortality events from mean 11.7 years of follow up, the longitudinal analysis strongly supports a temporal relationship between happiness and increased life expectancy. However, a tangible weakness of the study is the measurement of happiness (and covariates) were made at a single point in time only at baseline more than a decade ago, and not repeated at follow up to take into account possible changes occurring prior to death. Mood and emotional states change from time to time, and there may be mood fluctuations together with changes in physical and health status during the period prior to death. In relation to long-term mortality prediction, the stability or fluctuation of happiness (in line with changes in physical and health states) may be crucial. Studies suggest that the frequency and duration, rather than intensity, of happiness as a positive affect is more strongly related to well-being [12]. The frequency and duration of happiness over time was not measured and considered as a time-dependent covariate for mortality outcome in the Cox regression analysis. It was also not in the scope of the study to examine the effect of changes in socioeconomic development in the country on happiness and well-being. Further studies should also seek to better understand and disentangle the different constructs of positive psychological wellbeing including the positive affect construct of ‘happiness’ as well as life satisfaction influencing life expectancy. Additionally, studies that examine the complex relationship between socioeconomic development and happiness in the context of different cultural, contextual and individual factors are needed.

## Supplementary Information

**Additional file 1.** 

## Data Availability

The datasets used and/or analysed during the current study are available from the corresponding author on reasonable request.

## References

[1] Diener E (1984). Subjective well-being. Psychol Bull.

[2] Diener E, Chan MY (2011). Happy people live longer: subjective well-being contributes to health and longevity. Appl Psychol Health Well Being.

[3] Zaninotto P, Steptoe A (2019). Association between subjective well-being and living longer without disability or illness. JAMA Netw Open.

[4] Diener E (2017). If, why, and when subjective well-being influences health, and future needed research. Appl Psychol Health Well Being.

[5] Martín-María N (2017). The impact of subjective well-being on mortality: a meta-analysis of longitudinal studies in the general population. Psychosom Med.

[6] Xu G, Zhao C, Li Q (2021). Subjective well-being and mortality among the older people in China. China Population and Development Studies.

[7] Willroth EC (2020). Being happy and becoming happier as independent predictors of physical health and mortality. Psychosom Med.

[8] Härkänen T (2020). Estimating expected life-years and risk factor associations with mortality in Finland: cohort study. BMJ Open.

[9] Xu J, Roberts RE (2010). The power of positive emotions: It’sa matter of life or death—subjective well-being and longevity over 28 years in a general population. Health Psychol.

[10] Ostir GV (2000). Emotional well-being predicts subsequent functional independence and survival. J Am Geriatr Soc.

[11] Gana K (2016). Subjective wellbeing and longevity: findings from a 22-year cohort study. J Psychosom Res.

[12] Diener E, Oishi S, Lucas RE (2009). Subjective well-being: The science of happiness and life satisfaction. Oxford handbook of positive psychology.

[13] Boehm JK, Kubzansky LD (2012). The heart's content: the association between positive psychological well-being and cardiovascular health. Psychol Bull.

[14] Sin NL (2016). The protective role of positive well-being in cardiovascular disease: review of current evidence, mechanisms, and clinical implications. Curr Cardiol Rep.

[15] Pressman SD, Cohen S (2005). Does positive affect influence health?. Psychol Bull.

[16] Trudel-Fitzgerald C (2019). Prospective associations of happiness and optimism with lifestyle over up to two decades. Prev Med.

[17] Nabi, H., et al., Positive and negative affect and risk of coronary heart disease: Whitehall II prospective cohort study. Bmj, 2008;337(7660): a118. 10.1136/bmj.a118 PMC2443594 18595926

[18] Koopmans TA (2010). Effects of happiness on all-cause mortality during 15 years of follow-up: The Arnhem Elderly Study. Journal of Happiness Studies: An Interdisciplinary Forum on Subjective Well-Being.

[19] Chei C-L (2018). Happy older people live longer. Age Ageing.

[20] Liu B (2016). Does happiness itself directly affect mortality? The prospective UK Million Women Study. Lancet (London, England).

[21] Giltay EJ (2004). Dispositional optimism and all-cause and cardiovascular mortality in a prospective cohort of elderly dutch men and women. Arch Gen Psychiatry.

[22] Koivumaa-Honkanen H (2000). Self-reported life satisfaction and 20-year mortality in healthy Finnish adults. Am J Epidemiol.

[23] Lacruz ME (2011). Prospective association between self-reported life satisfaction and mortality: results from the MONICA/KORA Augsburg S3 survey cohort study. BMC Public Health.

[24] Shirai K (2009). Perceived level of life enjoyment and risks of cardiovascular disease incidence and mortality: the Japan public health center-based study. Circulation.

[25] Lu L, Gilmour R (2004). Culture and conceptions of happiness: individual oriented and social oriented swb. J Happiness Stud.

[26] Ngoo YT, Tey NP, Tan EC (2015). Determinants of life satisfaction in Asia. Soc Indic Res.

[27] Yang D, Zhou H (2017). The comparison between Chinese and Western Well-Being. Open J Soc Sci.

[28] Niti M (2008). Physical, social and productive leisure activities, cognitive decline and interaction with APOE-ε4 genotype in Chinese older adults. Int Psychogeriatr.

[29] Feng, L., et al., Metabolic syndrome and amnestic mild cognitive impairment: Singapore Longitudinal Ageing Study-2 findings. J Alzheimers Dis, 2013;34(3):649–57. 10.3233/JAD-121885 23246920

[30] Wei K (2017). Frailty and malnutrition: related and distinct syndrome prevalence and association among community-dwelling older adults: singapore longitudinal ageing studies. J Am Med Dir Assoc.

[31] Barger SD (2020). Is subjective well-being independently associated with mortality? a 14-year prospective cohort study in a representative sample of 25 139 US men and women. BMJ Open.

[32] Solé-Auró A (2018). Do women in Europe live longer and happier lives than men?. Eur J Pub Health.

[33] Lawrence EM, Rogers RG, Wadsworth T (1982). Happiness and longevity in the United States. Soc Sci Med.

[34] Ng TP (2016). Metabolic syndrome and the risk of mild cognitive impairment and progression to dementia: follow-up of the singapore longitudinal ageing study cohort. JAMA Neurol.

[35] Sheikh JI, Yesavage JA. Geriatric Depression Scale:recent evidence and development of a shorter version. Clin Gerontol. 1986;5:165–73. 10.1300/J018v05n01_09.

[36] Nyunt MS (2009). Criterion-based validity and reliability of the Geriatric Depression Screening Scale (GDS-15) in a large validation sample of community-living Asian older adults. Aging Ment Health.

[37] Rockwood K (2005). A global clinical measure of fitness and frailty in elderly people. CMAJ.

[38] Searle SD (2008). A standard procedure for creating a frailty index. BMC Geriatr.

[39] Cheong CY (2021). Physical and functional measures predicting long-term mortality in community-dwelling older adults: a comparative evaluation in the Singapore Longitudinal Ageing Study. Aging (Albany NY).

[40] Mitnitski AB (2002). Frailty, fitness and late-life mortality in relation to chronological and biological age. BMC Geriatr.

[41] Kimm H (2012). Life satisfaction and mortality in elderly people: the Kangwha Cohort Study. BMC Public Health.

[42] Tamosiunas A (2019). Psychological well-being and mortality: longitudinal findings from Lithuanian middle-aged and older adults study. Soc Psychiatry Psychiatr Epidemiol.

[43] Zaninotto P, Wardle J, Steptoe A (2016). Sustained enjoyment of life and mortality at older ages: analysis of the English Longitudinal Study of Ageing. BMJ.

[44] Jovanović, V. and M. Lazić, Is Longer Always Better? A Comparison of the Validity of Single-item Versus Multiple-item Measures of Life Satisfaction. Applied Research in Quality of Life, 2020; 15(3):675–92.

[45] Abdel-Khalek A (2006). Measuring happiness with a single-item scale. Soc Behav Personal Int J.

