# Supplementary material for: Happy people live longer because they are healthy people
Source: BMC Geriatr. 2023 Jul 18;23:440. doi: 10.1186/s12877-023-04030-w (PMC10354981; doi:10.1186/s12877-023-04030-w)
Supplement: Supplementary file 1 — Additional file 1. [file 12877_2023_4030_MOESM1_ESM.docx]

SUPPLEMENTARY TABLES

| **Table** S1. Estimates of correlation coefficients between physical health, psychological health, and happiness | | | | | | | | | | | | | | | | | |
| --- | --- | --- | --- | --- | --- | --- | --- | --- | --- | --- | --- | --- | --- | --- | --- | --- | --- |
| Variables | 1. | 2. | 3. | 4. | 5. | 6. | 7. | 8. | 9. | 10. | 11. | 12. | 13. | 14. | 15. | 16. | 17. |
| 1. Depression | 1.00 |  |  |  |  |  |  |  |  |  |  |  |  |  |  |  |  |
| 2. MCS score | -.35** | 1.00 |  |  |  |  |  |  |  |  |  |  |  |  |  |  |  |
| 3. Self-rated global health | -.17** | .19** | 1.00 |  |  |  |  |  |  |  |  |  |  |  |  |  |  |
| 4. Frailty index | .32** | -.36** | -.39** | 1.00 |  |  |  |  |  |  |  |  |  |  |  |  |  |
| 5. BMI | -.01 | -.002 | .02 | -.004 | 1.00 |  |  |  |  |  |  |  |  |  |  |  |  |
| 6. Diabetes | .04** | -.05** | -.12** | .33** | -.02 | 1.00 |  |  |  |  |  |  |  |  |  |  |  |
| 7. Heart disease | .09** | -.04** | -.15** | .35** | -.01 | .15** | 1.00 |  |  |  |  |  |  |  |  |  |  |
| 8. Stroke | .08** | -.05** | -.10** | .25** | -.02 | .09** | .52** | 1.00 |  |  |  |  |  |  |  |  |  |
| 9. CKD | .02 | -.03** | -.04** | .16** | -.004 | .07** | .14** | .09** | 1.00 |  |  |  |  |  |  |  |  |
| 10. COPD | .05** | -.04** | -.03 | .15** | -.009 | -.003 | .01 | .007 | .03 | 1.00 |  |  |  |  |  |  |  |
| 11. Arthritis | .05** | -.02 | -.09** | .20** | .002 | .03* | .03** | .000 | .02 | -.002 | 1.00 |  |  |  |  |  |  |
| 12. Mobility | -.001 | .01 | .02 | -.04** | -.004 | -.03* | .006 | .000 | -.004 | .02 | -.02 | 1.00 |  |  |  |  |  |
| 13. IADL dependency | .17** | -.13** | -.13** | .43** | .01 | .07** | .20** | .21** | .13** | .06** | .01 | -.02 | 1.00 |  |  |  |  |
| 14. PCS score | -.20** | .02 | .51** | -.55** | .007 | -.11** | -.20** | -.14** | -.09** | -.04** | -.17** | .03** | -.34** | 1.00 |  |  |  |
| 15. Multimorbidity | .11** | -.09** | -.23** | .60** | -.007 | .44** | .45** | .27** | .15** | .05** | .30** | -.03** | .17** | -.29** | 1.00 |  |  |
| 16. Happiness score | -.28** | .31** | .32** | -.24** | -.006 | -.05** | -.08** | -.05** | -.006 | -.04** | -.05** | -.009 | -.11** | -.23** | -.10** | 1.00 |  |
| 17. Happiness categories | -.31** | .29** | .24** | -.24** | .004 | -.05** | -.09** | -.06** | -.007 | -.04* | -.03* | -.01 | -.11** | .22** | -.11** | .83** | 1.00 |
|  |  |  |  |  |  |  |  |  |  |  |  |  |  |  |  |  |  |
| *Note*: **p* < .05, ***p*< .01 | | | | | | | | | | | | | | | | | |
